# The sugar-free paradox: cardiometabolic consequences of erythritol

**DOI:** 10.1038/s41392-023-01504-6

**Published:** 2023-06-16

**Authors:** Kalliopi Pafili, Michael Roden

**Affiliations:** 1 Department of Endocrinology and Diabetology, Medical Faculty and University Hospital Düsseldorf, Heinrich-Heine-University Düsseldorf, 40225 Düsseldorf, Germany; 2 German Center for Diabetes Research, Partner Düsseldorf, 85764 München-Neuherberg, Germany; 3 Institute for Clinical Diabetology, German Diabetes Center, Leibniz Center for Diabetes Research at Heinrich-Heine-University Düsseldorf, 40225 Düsseldorf, Germany

**Keywords:** Cell biology, Endocrine system and metabolic diseases, Cardiology, Outcomes research, Cardiovascular diseases

In a recent study published in Nature Medicine, Witkowski and colleagues applied untargeted metabolomics studies in 1157 people at high risk for cardiovascular disease, comprising 22% with diabetes mellitus, followed by quantitative targeted analyses to identify circulating metabolites associated with an increased cardiovascular risk.^1^ Their analyses revealed an association between plasma erythritol and major cardiovascular events—defined as the composite of death, myocardial infarction, and stroke during a follow-up period of 3 years.^1^

**Fig. 1:**
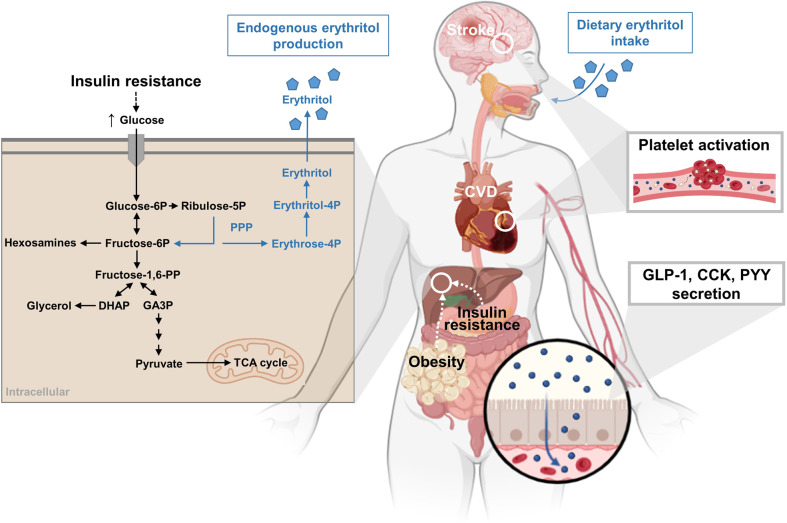
The non-nutritive sweetener, erythritol, induces platelet activation and aggregation and may promote clot formation in people at high cardiovascular risk who exhibit a positive association between plasma erythritol and cardiovascular outcomes.^1^ Of note, elevated plasma erythritol concentrations arise from both dietary intake and from endogenous production in erythrocytes, liver, and kidney.^4^ Erythritol may predict central adiposity and insulin resistance^1^ but also stimulate gastrointestinal hormone secretion.^5^ Dash arrows refer to potential bidirectional relationships. CCK cholecystokinin, CVD cardiovascular disease, DHAP dihydroxyacetonphosphate, GA3P glyceraldehyde 3-phosphate, GLP-1 glucagon-like peptide-1, P phosphate, PP biphosphate, PPP pentose phosphate pathway, PYY polypeptide YY, TCA tricarboxylic acid cycle. Cartoons were created with BioRender.com

Although seemingly unexpected, these findings support previous metabolomic analyses of the Atherosclerosis Risk in Communities (ARIC) cohort, which demonstrated a significant albeit smaller increase in the hazard ratio of incident coronary heart disease for serum erythritol concentrations, also occurring independently of body mass.^2^ Consumption of some other non-nutritive sweeteners (aspartame, acesulfame potassium, sucralose) has already been associated with excess risk of cardiovascular disease, including cerebrovascular events and coronary heart disease.^3^ This apparent sugar-free paradox underlying the artificial sweetener-related cardiometabolic risk could result from direct effects but also from metabolic alterations such as central adiposity, hypertriglyceridemia, insulin resistance, and type 2 diabetes (Fig. 1),^1,3^ although effects on weight gain remain controversial.^3^

Erythritol, a naturally occurring sugar alcohol without significant caloric value, is rapidly absorbed upon ingestion from the small intestine via passive diffusion (Fig. 1).^4^ Additionally, erythritol is endogenously produced via the pentose phosphate pathway (Fig. 1),^1^ also termed hexose monophosphate shunt, which generates, among others, erythrose-4-phosphate required for aromatic amino acid synthesis. Studies confirmed the endogenous erythritol production in human cells, including erythrocytes, and suggested metabolically active tissues, i.e., liver and kidney, as the main contributors to systemic erythritol levels.^4^ Naturally, excessive glucose and fructose availability, typical for obesity and insulin resistance, likely drives erythritol production and its systemic overflow (Fig. 1). Accordingly, plasma erythritol elevation has been linked to central adiposity and type 2 diabetes^1^ but also hyperglycemia-related nephropathy and retinopathy.^4^ Moreover, mice receiving tap water, supplemented by 4% erythritol for 4 weeks, showed expanded white adipose tissue, and decreased oxygen consumption, along with increased liver and muscle lipid content and glucose intolerance.^4^ On the other hand, acute 10–50 g erythritol administration for up to 24 h revealed rather beneficial effects, such as dose-dependent stimulation of anorexigenic hormones (glucagon-like peptide-1 (GLP-1), cholecystokinin (CCK), peptide YY (PYY)) delaying gastric emptying, but without impact on glycemia, lipidemia, insulinemia, glucagonemia or glucose-dependent insulinotropic polypeptide (GIP) in double-blind, randomized controlled trials of healthy humans.^5^ One might hypothesize that the described mechanisms reflect a mutual interaction of both exogenous and endogenous erythritol accumulation with central adiposity and insulin resistance, ultimately increasing cardiovascular risk.

Abnormal vascular function and coagulation represent other central drivers of cardiovascular disease onset and development.^3^ Witkowski and colleagues^1^ provided both in vitro and in vivo evidence for the effects of erythritol on platelet function and thrombosis potential. They validated their initial findings by targeted metabolomic analyses in two different cohorts with similarly increased cardiovascular risk, from the United States (*n* = 2149) and Europe (*n* = 833) undergoing cardiac evaluation. This follow-up analysis strengthened their original findings by revealing that the association of erythritol not only remained significant but was independent of age, sex, and other established risk factors. This suggests the additional operation of other than metabolic mechanisms. Indeed both in vitro and in vivo studies in human whole blood and mice revealed an erythritol-elicited increase in platelet adhesion and clot formation (Fig. 1).^1^ Their subsequent pilot study showed that a single dose of 30 g erythritol results in prolonged excessive plasma erythritol elevation for over 48 h. The recorded concentrations remained well above the threshold, which increased platelet reactivity and thrombocyte reactivity in vitro,^1^ suggesting a prolonged thrombotic risk arising from exogenous erythritol.

While these studies support a novel conceptual framework of erythritol-induced promotion of thrombus formation leading to excess cardiovascular risk, several limitations need to be addressed. There is not only a lack of standardization of erythritol measurements but also of the preanalytical conditions. The observed prolonged elevation of erythritol upon dietary intake raises the question of whether simple overnight fasting conditions suffice for the interpretation of baseline concentrations; moreover, the kinetics of erythritol elimination might substantially differ between healthy and metabolically compromised people. Importantly, their in vitro procedures raised the platelet concentrations creating artificial experimental conditions. Also, the mice experiments showing enhanced clot formation upon erythritol injection do not necessarily mirror thrombosis risk in humans. Thus, a direct analysis of platelet activation in humans upon erythritol might better reflect real-life conditions. Further questions arise: to what extent do different conditions affect the relative contributions of exogenous versus endogenous sources to the total circulating erythritol concentration, particularly could people with increased cardiovascular risk produce more erythritol? One might speculate that altered hepatic metabolism in humans with type 2 diabetes, who are at higher risk of fatty liver disease, might at least contribute to the described effects. Of note, epidemiological and cohort studies cannot exclude reverse causality because people with an unfavorable metabolic profile are more prone to consume sugar-free meals to avoid weight gain or hyperglycemia, even before cardiovascular disease diagnosis.^3^ At present, controlled trials did not detect detrimental effects of erythritol ingestion in healthy humans,^5^ but specific trials on vascular function and coagulation in healthy and diseased humans are lacking.

Taken together, Witkowski and colleagues shed light on one of the widely-used non-nutritive sweeteners and provide novel evidence for the potentially deleterious effects of erythritol on platelet function. At the same time, these findings raise new questions calling for sufficiently powered double-blind, randomized controlled trials addressing the safety as well as mechanistic studies examining the Janus-head-like features of erythritol. Importantly, the evidence derived from the current study should already suffice for the authorities to re-evaluate their policy statements regarding the cardiovascular and metabolic risks of artificial sweeteners.
